# Assessment of SARC-F Sensitivity for Probable Sarcopenia Among Community-Dwelling Older Adults: Cross-Sectional Questionnaire Study

**DOI:** 10.2196/54475

**Published:** 2025-07-25

**Authors:** David Propst, Lauren Biscardi, Tim Dornemann

**Affiliations:** 1 Department of Exercise Science, School of Health Sciences, Barton College, 200 Acc Dr W, Wilson, NC, 27893, United States, 1 252-864-6242; 2 Department of Exercise Science, School of Health Sciences, North Carolina Wesleyan College, Rocky Mount, NC, United States

**Keywords:** sarcopenia, neuromuscular, screening, community, scale, measure, questionnaires, diagnosis, gerontology, older adults, muscular

## Abstract

**Background:**

The European Working Group on Sarcopenia in Older People (EWGSOP2) recommends the use of the 5-item SARC-F (strength, assistance with walking, rising from a chair, climbing stairs, and falls) questionnaire by clinicians to screen for probable sarcopenia. The recommended threshold of ≥4 has low sensitivity and high specificity in identifying probable sarcopenia. While this high threshold is effective in excluding clients without probable sarcopenia, challenges exist in using this screening tool to identify clients with low muscle strength.

**Objective:**

This study aims to reassess the use of SARC-F in a primary care clinic for the determination of incidence of probable sarcopenia and to evaluate if a handgrip strength test is necessary for its diagnosis.

**Methods:**

We screened 204 patients aged ≥65 years (117 men and 87 women) during routine visits with the SARC-F questionnaire. Probable sarcopenia was defined by EWGSOP2 grip strength cut points (≤27 kg for men and ≤16 kg for women). Receiver operating characteristic analysis was performed to identify the SARC-F threshold that best balanced sensitivity and specificity.

**Results:**

Probable sarcopenia was present in 12% (n=24) of participants. The mean age (73.9, SD 6.2 years) and mean BMI (29.5, SD 5.8 kg/m²) did not differ significantly by sex; however, men showed a higher mean grip strength (36.3, SD 8.1 kg vs 22.4, SD 5.5 kg; *P*<.001) and lower mean SARC-F scores (0.9, SD  1.7 vs 1.9, SD 2.3; *P*<.001). A SARC-F cut point of ≥2 yielded an area under the curve of 0.77 (95% CI 0.67‐0.88), with sensitivity of 0.78, specificity of 0.75, accuracy of 0.77, positive predictive value of 0.31, and negative predictive value of 0.96. The grip strength differed significantly between screen-positive and screen-negative groups at both the ≥2 and ≥4 thresholds (*P*<.001).

**Conclusions:**

A SARC- F threshold of ≥2 is recommended as an optimal trade-off between sensitivity and specificity for identifying community-dwelling older adults with probable sarcopenia. This threshold is lower than the currently accepted recommendation of ≥4. Our findings promote the recommendations for early detection and treatment by medical professionals following the EWGSOP2 by improving the ability of clinicians to identify individuals with low muscle strength using this screening procedure.

## Introduction

Sarcopenia has been defined as a progressive loss of muscle mass and strength that adversely affects mobility, function, fall risk, and mortality in older adults [1-3]. Age-related muscle and strength loss can begin as early as 30 years of age and accelerate after 50 years of age [3-5]. The severity of muscle mass and strength loss in sarcopenia has been shown to be associated with a decreased ability to complete activities of daily living, lower quality of life, and substantially higher health care costs [5,6].

In 2018, the second European Working Group on Sarcopenia in Older People (EWGSOP2) defined a multifactorial approach to identifying sarcopenia by finding, assessing, confirming, and testing for severity [6]. This model initially screens for sarcopenia through the use of strength, assistance with walking, rising from a chair, climbing stairs, and falls through use of a clinical symptom index (eg, SARC-F [strength, assistance with walking, rising from a chair, climbing stairs, and falls]) questionnaire or using clinical suspicion [6,7].

Individuals that are identified as potentially having sarcopenia through screening undergo a muscular strength test. If strength levels meet the criteria for sarcopenia, muscle quality testing is conducted to confirm the diagnosis [3,6]. Next, the severity of sarcopenia is determined using a physical performance test [3,6].

Despite Rosenberg [8] coining the term “sarcopenia” in 1989 and the development of the *ICD-10* code *M62.84* in 2016 [9], a recent survey found that only 20% of doctors are aware of sarcopenia, a condition that can lead to falls, fractures, disability, and chronic diseases [10]. If physicians are not aware of sarcopenia, they may not screen for it or diagnose it correctly. This can lead to delays in treatment, which can have serious consequences for patients.

Early detection of sarcopenia through screening programs is crucial, as evidenced by research demonstrating that screening can lead to increased quality-adjusted life years and improved health outcomes for older adults [11,12].

While research has been conducted on various aspects of sarcopenia, including its prevalence, risk factors, and health outcomes, there has been limited focus on the practical challenges of managing this condition in primary care settings. This gap in the literature is concerning, given that primary care serves as the first point of contact for patients and plays a crucial role in early detection and management of sarcopenia [12]. Diagnosis of sarcopenia requires muscle strength testing, muscle quality testing, and a physical performance test, which is not practical in a primary care clinic. A recent review by Porter et al [13] found that primary care providers were estimated to require 26.7 hours per day, comprising 14.1 hours per day for preventive care, 7.2 hours per day for chronic disease care, 2.2 hours per day for acute care, and 3.2 hours per day for documentation and inbox management. Therefore, any additional screening must demonstrate accuracy along with being both time-efficient and cost-effective.

The EWGSOP2 pathway classifies patients as having probable sarcopenia when a brief symptom screen (eg, SARC-F) is followed by objectively low muscle strength (ie, grip or chair-stand) [6]. To embed this approach in routine care, our clinic now screens every patient aged ≥65 years during the annual physical examination. Using these real-world data, we posed two questions: (1) How common is probable sarcopenia in our practice? and (2) Can the SARC-F alone, with an optimized cut-point, serve as an efficient first-line screen? Prior studies have linked SARC-F to grip strength but did not validate a lower threshold in primary care.

## Methods

A total of 204 community-dwelling older adults (ie, 87 female and 117 males) 65 years or older were screened during their regularly scheduled physician visits. Participants completed a SARC-F questionnaire and a grip strength assessment. Participant demographic data including age, gender, and BMI were recorded.

### Inclusion and Exclusion Criteria

Community-dwelling adults  aged ≥65 years who attended routine primary care appointments between November 2022 and March 2023 and were able to complete the SARC-F questionnaire and the 3-trial dominant-hand grip-strength test were eligible for inclusion. Patients were excluded if acute illness, recent upper-limb injury, severe arthritis, neurologic disease, or marked cognitive impairment precluded safe grip testing or questionnaire completion. These criteria reflect pragmatic screening practices and maximize both patient safety and data validity.

### SARC-F Questionnaire

The SARC-F was selected as the screening tool of interest in this study. The SARC-F is a five question self-report survey developed by Malmstrom et al [7] to detect clinical symptoms of sarcopenia. The SARC-F questions include asking the patients to report difficulties with strength, assistance walking, rising from a chair, climbing stairs, and falls. The first four items are scored as 0 (no difficulty), 1 (some difficulty), or 2 (a lot of difficulty). Number of falls in the past year is rated as 0 (no falls), 1 (between 1‐3 falls), or 2 (4 or more falls). The sensitivity is low to moderate, and the specificity is high to predict low muscle strength when a cutoff value of ≥4 is used.

### Grip Strength

Muscle strength is the criterion used to detect probable sarcopenia in clinical settings [6]. Grip strength was selected as the measure of skeletal muscle strength because it is a quick and easy tool to administer during physician visits. Diagnosis of probable sarcopenia was assessed using the gender-specific recommended cutoff values for grip strength by the EWGSOP2 [6,14]. These values are <27 kg for men and <16 kg for women [14]. All grip tests were performed in private exam rooms by the first author. Participants sat with elbows flexed at 90°, wrists in a neutral position, and feet flat. Using a calibrated digital dynamometer (Sutekus Digital), each participant performed three maximal efforts (3‐5 s) with 30‐60 seconds of rest. The highest value for the dominant hand was used for analysis.

### Ethical Considerations

This study was approved by the Barton College Institutional Review Board (IRB #2022000034; approval date  January 25,  2023). As SARC-F screening and grip-strength testing are standard components of routine visits for adults 65 years or older at the study clinic, informed consent was not required as the data were obtained from deidentified medical records in accordance with the Health Insurance Portability and Accountability Act. All patient information was anonymized prior to analysis to ensure confidentiality. The collected data were anonymized, and no compensation was provided to participants.

### Data Collection and Statistical Analysis

Deidentified encounter records supplied data on age, sex, BMI, SARC-F score, and dominant-hand grip strength. Normality was assessed using Kolmogorov-Smirnov tests and histograms. Between-group differences were analyzed with independent 2-tailed *t* tests (parametric) or Mann-Whitney *U* tests (nonparametric). Receiver operating characteristic (ROC) analysis evaluated the ability of SARC-F to detect probable sarcopenia (EWGSOP2 grip-strength thresholds) and generated area under the curve (AUC) estimates with 95% CIs. Sensitivity, specificity, predictive values, and accuracy were calculated at cut points 2 and 4. Effect sizes (Cohen *d* or *r*) quantified the magnitude of differences. Post hoc power for the ROC (n=204; AUC=0.75) was 98.6%.

A ROC curve was used to determine a threshold (SARC-F score) that optimized the balance between sensitivity and specificity for diagnosing probable sarcopenia. The AUC was calculated to present the ability of the SARC-F score to discriminate between probable sarcopenic and nonsarcopenic individuals. An AUC of 1.0 indicates perfect discrimination capability, 0.5 indicates discrimination capability equal to that of chance, and 0.0 indicates that all subjects are incorrectly classified.

Sensitivity, specificity, positive predictive value, negative predictive value, and false positive rate were calculated for SARC-F threshold scores. Sensitivity was calculated as the number of participants diagnosed with probable sarcopenia that were correctly identified by the SARC-F screening. Specificity was calculated as the number of participants not diagnosed with probable sarcopenia that correctly screened negative with the SARC-F. Positive predictive value was calculated as the number of participants diagnosed with probable sarcopenia that screened positive with the SARC-F. Negative predictive value was calculated as the number of participants without probable sarcopenia that screened negative with the SARC-F. The false positive rate was calculated as the ratio of the number of participants screened positive by the SARC-F without probable sarcopenia to the number of participants who were not diagnosed with probable sarcopenia. Accuracy was also calculated at each SARC-F threshold as the proportion of correctly classified patients (both true positives and true negatives).

Comparisons of muscle strength between groups determined by the SARC-F threshold of 2 and previously recommended SARC-F threshold of 4 were performed, following the between-group comparison procedures listed above. When variances were not equal, Welch *t* test was used. The α was set at .05. All statistical analyses were performed using RStudio software (version 2023.06.1; Posit PBC).

## Results

### SARC-F Questionnaire Scores

**Figure 1.**
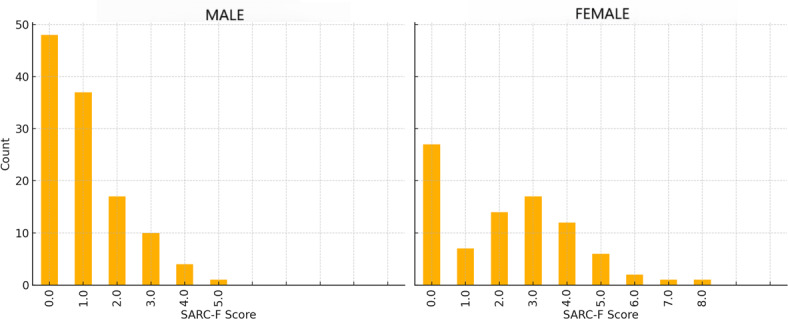
Distribution of SARC-F scores by sex. Histograms show score frequencies for male (left) and female participants (right), respectively. SARC-F: strength, assistance with walking, rising from a chair, climbing stairs, and falls.

Probable sarcopenia was present in 12% (n=24) of participants. Participant characteristics for age, BMI, grip strength, and SARC-F score are presented in Table 1. There was a significant difference in grip strength between men and women (*t*_99.51_=−14.25; *P*<.001; *d*=1.95) and SARC-F score (*U*=6307; *P*<.001; *r*=0.24). The sex-specific distribution of SARC-F scores is illustrated in Figure 1. There was no significant difference between BMI of men and women (*t*_145.3_=1.39; *P*=.17) or age (*t*_201_=−0.134; *P*=.89).

**Table 1.** Participant characteristics (N=204).

Variables	Overall (N=204), mean (SD)	Women (n=87), median (SD)	Men (n=117), median (SD)	*P* value
Age (years)	73.9 (6.2)	73.8 (5.9)	73.9 (6.4)	.89
BMI (kg/m^2^)	29.5 (5.8)	30.2 (6.8)	29.0 (4.8)	.17
Grip strength (kg)	30.4 (9.9)	22.4 (5.5)	36.3 (8.1)	<.001^a^
SARC-F^b^ score	1.33 (2.01)	1.88 (2.31)	0.92 (1.65)	<.001^a^

a Denotes significant difference from females (*P*<.001).

b SARC-F: strength, assistance with walking, rising from a chair, climbing stairs, and falls.

**Figure 2.**
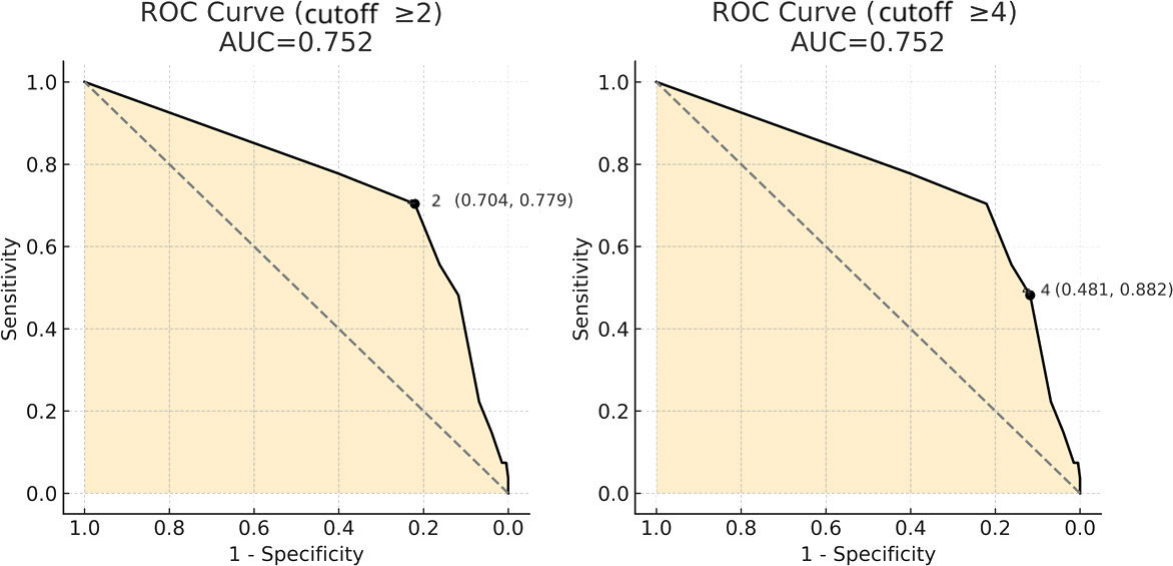
Combined ROC curves for SARC-F thresholds  ≥2 and ≥4. AUC: area under the curve; ROC: receiver operating characteristic; SARC-F: strength, assistance with walking, rising from a chair, climbing stairs, and falls.

Figure 2 presents the combined ROC curve for thresholds ≥2 and ≥4. The AUC for both thresholds was 0.752 (95% CI 0.66-0.84). We compared the diagnostic performance of SARC-F across the two commonly used cut points (≥2 vs ≥4). Using DeLong test for paired ROC curves, the AUCs were not significantly different (AUC  0.752 vs 0.752; *P*=.98), supporting the clinical preference for the more sensitive ≥2 threshold. A post hoc power analysis for the ROC curve revealed statistical power of 99.5%. Calculations for sensitivity, specificity, positive predictive value, negative predictive value, false positive rate, and accuracy for SARC-F cutoff scores are presented in Table 2.

**Table 2.** Diagnostic operating characteristics at SARC-F^a^ thresholds.

Cutoff values	Sensitivity (95% CI)	Specificity (95% CI)	FPR^b^	PPV^c^	NPV^d^	Accuracy
0	1.00 (1.00‐1.00)	0.00 (0.00‐0.00)	1.00	0.12	1.00	0.12
1	0.79 (0.63‐0.96)	0.60 (0.53‐0.67)	0.40	0.21	1.00	0.62
2	0.75 (0.58‐0.92)	0.78 (0.72‐0.83)	0.22	0.31	0.96	0.77
3	0.63 (0.42‐0.83)	0.83 (0.78‐0.88)	0.17	0.33	0.94	0.81
4	0.58 (0.38‐0.75)	0.88 (0.83‐0.93)	0.12	0.40	0.94	0.85
5	0.29 (0.13‐0.46)	0.93 (0.89‐0.96)	0.07	0.35	0.91	0.85
6	0.17 (0.04‐0.33)	0.96 (0.96‐0.98)	0.04	0.33	0.90	0.86
7	0.08 (0.00‐0.21)	0.98 (0.96‐1.00)	0.02	0.40	0.89	0.88
8	0.08 (0.00‐0.21)	0.99 (0.98‐1.00)	0.01	0.67	0.89	0.89

a SARC-F: strength, assistance with walking, rising from a chair, climbing stairs, and falls.

b FPR: false positive rate.

c PPV: positive predictive value.

d NPV: negative predictive value.

### Grip Strength

Using a SARC-F cut point of  ≥2, participants classified as having probable sarcopenia (n=64) had higher SARC-F scores (*t*_59_=16.7; *P*<.001), were older (*t*_80_=3.3; *P*=.001), had higher BMI (*t*_76.9_= 2.7; *P*=.009), and demonstrated lower grip strength (*t*_121_=8.0; *P*<.001) than those with SARC-F <2. Using a cut point of  ≥4, SARC-F scores and grip strength differed significantly (*t*_66.9_= 7.8; *P*<.001), whereas age and BMI were similar (*P*=.05 and *P=*.06, respectively). Mean values are summarized in Table 3.

**Table 3.** Comparison of demographic and strength variables by SARC-F threshold.

SARC-F^a^ threshold	Participants, n	SARC-F, mean (SD)	Age (years), mean (SD)	BMI (kg/m²), mean (SD)	Grip strength (kg), mean (SD)
<2	167	0.23 (0.4)	72.8 (5.3)	28.9 (4.8)	33.5 (9.2)
≥2	64	3.97 (1.8)	76.7 (7.4)	31.4 (7.4)	23.5 (7.7)
<4	194	0.54 (0.9)	73.3 (5.7)	29.1 (5.3)	32.4 (9.6)
≥4	37	5.11 (1.4)	76.9 (7.9)	32.0 (7.3)	22.0 (6.4)

a SARC-F: strength, assistance with walking, rising from a chair, climbing stairs, and falls.

The composite ROC curve for the two cut points is presented in Figure 2. The AUC for both thresholds was 0.752 (95% CI 0.66‐0.84). DeLong test showed no significant difference between AUCs (*P*=.98), supporting the clinical use of the more sensitive ≥2 threshold. Post hoc power for the ROC analysis was 99.5%. Diagnostic operating characteristics for each threshold are provided in Table 2.

## Discussion

### Principal Findings

A SARC-F cut point of ≥2 balanced sensitivity and specificity better than the traditional ≥4 threshold, identifying probable sarcopenia in 31% (n=63) of community-dwelling adults 65 years or older without adding clinic burden. Men demonstrated higher grip strength and lower SARC-F scores than women, reaffirming sex-specific muscle-strength disparities.

### Comparison With Prior Work

Earlier studies reported high specificity but modest sensitivity when applying a SARC-F  ≥4 [6,7,15]; our findings replicated this pattern (58% sensitivity, 88% specificity) while confirming that lowering the threshold to  ≥2 improves case finding (78% sensitivity) while maintaining acceptable specificity (75%). Our AUC of 0.75 aligns with the AUC of 0.71 as reported by Erbas Sacar et al [16], supporting the tool’s value as a screening and not a stand-alone diagnostic test. Recent authors have advocated thresholds as low as  ≥1 for maximal sensitivity [14,16,17]; our operating characteristic table (Table 2) illustrates the same trade-off: as the cut point increases, sensitivity decreases and specificity increases. DeLong test showed no difference between AUCs for the two thresholds (*P*=.98), strengthening the argument for the more sensitive ≥2 cut point in primary care.

### Strengths and Limitations

First, real-world implementation during annual visits increases external validity. Second, standardized grip-strength testing minimized measurement error. Third, the sample (N=204) provided 98.6% post hoc power for ROC analyses. Limitations included (1) the single-site, cross-sectional design limits generalizability and causal inference; (2) SARC-F relies on self-report and may incur recall bias; (3) potential confounders (physical activity, cognitive status, comorbidities) were not captured; and (4) grip strength was measured once, and functional measures such as gait speed were unavailable. Each factor may attenuate or inflate the observed associations, underscoring the need for multimodal assessment in future research.

### Future Directions

Prospective, multicenter studies should validate the ≥2 threshold across diverse settings, incorporate additional functional tests, and examine longitudinal outcomes (ie, falls, hospitalization, disability). Cost-effectiveness analyses could further justify routine SARC-F screening in primary care, and digital integration of the questionnaire into electronic health records may streamline population-level implementation.

### Conclusion

A SARC-F cut point of  ≥2 offers a feasible, time-efficient approach to flag older primary care patients who require confirmatory strength testing, aligning with EWGSOP2 recommendations for early clinical intervention. The tool should be used to complement—rather than replace—comprehensive diagnostic workups.

## Abbreviations

AUC: area under the curve
EWGSOP2: The European Working Group on Sarcopenia in Older People
ROC: receiver operating characteristic
SARC-F: strength, assistance with walking, rising from a chair, climbing stairs, and falls
